# A Fulminant Case of Acute Respiratory Distress Syndrome Associated with Mycoplasma Pneumonia Treated with Nasal High-Flow Oxygen Therapy

**DOI:** 10.1155/2018/1067593

**Published:** 2018-10-21

**Authors:** Naoki Kawakami, Ho Namkoong, Takanori Ohata, Shinji Sakaguchi, Fumitake Saito, Hideki Yuki

**Affiliations:** ^1^Department of Emergency and Critical Care Medicine, St. Luke's International Hospital, 9-1 Akashicho, Chuo-ku, Tokyo 104-8560, Japan; ^2^Department of Pulmonary Medicine, Eiju General Hospital, 2-23-16 Higashiueno, Taito-ku, Tokyo 110-8645, Japan; ^3^Division of Pulmonary Medicine, Department of Medicine, Keio University School of Medicine, 35 Shinanomachi, Shinjuku-ku, Tokyo 160-8582, Japan; ^4^Laboratory of Clinical Immunology and Microbiology, National Institute of Allergy and Infectious Diseases, NIH, Bethesda, MD. 20814, USA

## Abstract

**Introduction:**

The prognosis of mycoplasma pneumonia in adults is generally favorable, but a few patients show progression to acute respiratory distress syndrome (ARDS). We have described the management of a patient who showed progression of mycoplasma pneumonia to ARDS.

**Presentation of Case:**

A 26-year-old male patient with no significant past medical or social history presented with a 5-day history of fever. Following this, he was diagnosed with bacterial pneumonia and treated with tazobactam/piperacillin; however, he showed little clinical improvement with this treatment approach. We diagnosed the patient with mycoplasma pneumonia with an antigen test and treated him with azithromycin and prednisolone. Despite the appropriate antimicrobial therapy, his symptoms worsened and therefore we changed his oxygen therapy from a reservoir mask to nasal high-flow oxygen in addition to minocycline. Consequently, with this treatment, he recovered from severe mycoplasma pneumonia.

**Discussion:**

In patients with severe pneumonia who experience respiratory failure, it has been reported that nasal high-flow oxygen therapy is not inferior to noninvasive positive pressure ventilation therapy regarding intubation rate. In this case, induction of nasal high-flow oxygen therapy led to avoidance of ventilator management. This is a valuable case report highlighting the optimal outcome of nasal high-flow oxygen therapy in a fulminant case of acute respiratory distress syndrome.

**Conclusion:**

In patients who present with severe mycoplasma pneumonia with respiratory failure, nasal high-flow oxygen therapy can help reduce the needs for ventilator management including intubation.

## 1. Introduction

Pneumonia is a major cause of morbidity and mortality worldwide [1]. Approximately one-sixth of patients hospitalized with community acquired pneumonia (CAP) are admitted to the ICU, and among these patients, half of them required mechanical ventilation [2].

Although the prognosis of mycoplasma pneumonia in adults is generally fair, there are 5.6% of cases associated with respiratory failure, and it is reported that all of them required ventilator management [3]. We describe the management of a patient with mycoplasma pneumonia who experienced respiratory distress and required nasal high-flow oxygen in addition to conventional antibiotic therapy.

## 2. Case

A 26-year-old male patient with no remarkable medical and social history presented with a five-day history of a cough and fever. Before coming to our hospital, he went to a clinic and received amoxicillin, which resulted in no improvement of his symptoms. He visited another hospital three days before presenting to us and was hospitalized with a diagnosis of bacterial pneumonia. Although he received tazobactam/piperacillin, his clinical symptoms showed deterioration. Two days later, he was admitted to our hospital.

His vital signs were as follows: temperature, 38.1°C, heart rate of 112 beats/min, blood pressure of 98/60* *mmHg, respiratory rate of 24 breaths/min, and saturation of peripheral oxygen of 94% with a reservoir mask of 6 L/min. On physical examination, weak respiratory sounds and coarse crackles were heard in the lower left chest.

His initial white blood cell count was 6,800/*μ*L, hemoglobin was 14.0g/dL, platelet count was 133,000/*μ*L, and C-reactive protein level was 34.30 mg/dL (Table 1). Arterial blood gas (reservoir mask of 6 L/min) showed that the pH was 7.44, PCO_2_ was 41.1 mmHg, PO_2_ was 69.2 mmHg, HCO_3_^−^ was 27.2 mmol/l, and BE was 2.8 mmol/L. Among the viral and bacterial rapid tests performed, only the Mycoplasma antigen tested positive.

**Figure 1:**
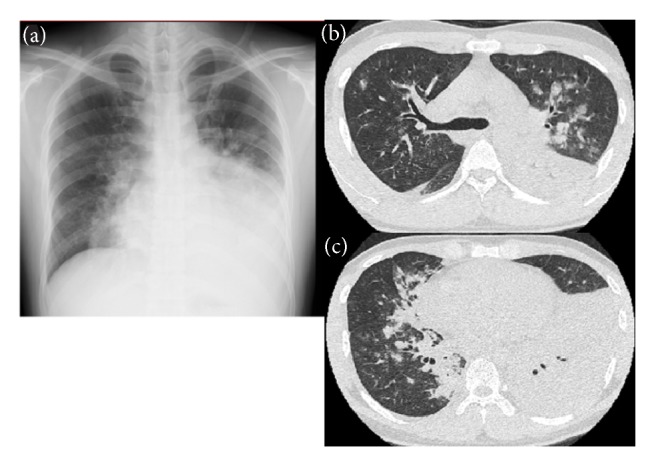
Chest radiography and chest computed tomography findings. (a) Chest radiography taken at the time of hospitalization, showing an infiltrating shadow in left middle lung field and right lower lung field. (b)-(c) Computed tomography scan taken at the time of hospitalization, showing lobar pneumonia in the lower left lobe and infiltrating shadow in the left whole lung lobe and the lower right lobe.

Chest radiography indicated an infiltrating shadow in left middle lung field and right lower lung field. A chest CT indicated lobar pneumonia in the lower left lobe and an infiltrating shadow in the left whole lung lobe and the lower right lobe (Figure 1). Based on these findings, the patient was diagnosed with severe mycoplasma pneumonia.

Azithromycin 500 mg/day and prednisolone 30 mg/day were initiated. After admission, his respiratory condition worsened until a reservoir mask 15 L/min was required. We then changed the reservoir mask to a nasal high-flow oxygen (40 L/min, FiO_2_:0.8) and increased the prednisolone to 60 mg/day. In case of macrolide resistance, we additionally administrated minocycline 200 mg/day. On hospital day 4, the patient's respiratory status and inflammatory markers on laboratory findings improved. On hospital day 20, he was discharged.

## 3. Discussion

Nasal high-flow oxygen therapy, which allows the delivery of heated and humidified oxygen at a high- flow rate, can accurately adjust the inhaled oxygen concentration [4]. The high-flow oxygen also washes out nasopharyngeal dead space and reduces upper airway resistance by positive distending pressure. This mechanism can help support breathing and decrease ventilation-perfusion mismatches in the lung [5]. Overall, the high-flow approach results in oxygenation improvement.

In patients with acute hypoxemic respiratory failure, an intubation rate of 38% in the high-flow oxygen group and 50% in the noninvasive ventilation group has been reported. This showed that treatment with high-flow oxygen or noninvasive ventilation did not result in significantly different intubation rates [6]. In this case, we administrated nasal high-flow oxygen therapy, which prevented the need for ventilator management. Considering that mycoplasma pneumonia is a self-limiting disease, nasal high-flow oxygen therapy can be a good indication for those with severe mycoplasma pneumonia.

It has been reported in a study comparing the acute use of methylprednisolone with that of placebo that, among patients with severe community-acquired pneumonia and high initial inflammatory response, there was a decreased need for invasive mechanical ventilation not present at baseline [7]. In this case, the addition of prednisolone might also help avoid ventilator management.

Macrolide resistant bacteria is rapidly increasing in conjunction with the usage of macrolide antibiotics in Japan, and the macrolide resistance rate increased from 10% to 35% in adults from 2008 to 2011 [8]. Of 107 mycoplasma pneumonia cases in our area including this case, 52% of patients tested positive for macrolide resistant mycoplasma bacteria [9]. Later, it was reported that our patient had macrolide resistant mycoplasma bacteria.

The limitation of this study was that we could not identify which intervention, among minocycline, prednisolone, and HFNC, contributed to the improvement of the patient's respiratory status because they were all performed almost simultaneously.

In conclusion, it is important that the introduction of multidisciplinary treatment including nasal high-flow oxygen therapy for patients with severe mycoplasma pneumonia is taken into consideration to avoid ventilator management.

## Figures and Tables

**Table 1:** Blood test findings.

Item	Result
White blood cells (/*μ*L)	6,800

Hemoglobin (g/dL)	14.0

Platelets (/*μ*L)	133,000

C-reactive protein (mg/dL)	34.30

Aspartate transaminase (U/L)	79

Alanine transaminase (U/L)	38

Lactate dehydrogenase (U/L)	393

Creatine Phosphokinase (mg/dL)	307

Fe(*µ*g/dL)	20

Ferritin(ng/mL)	1924
